# Hydrogen bonds or not? Synthesis and structure of 2,3-di­cyanona­phthalene-1,4-diyl bis­(4-methylbenzene-1-sulfonate)

**DOI:** 10.1107/S2056989026002884

**Published:** 2026-03-24

**Authors:** Nesuhi Akdemir, Muhammad Nawaz Tahir, Muhammad Ashfaq

**Affiliations:** a Amasya University, Art and Science Faculty, Department of Chemistry, 05100, İpekköy, AMASYA, Türkiye; b Department of Physics, University of Sargodha, Sargodha 40100, Punjab, Pakistan; University of Aberdeen, United Kingdom

**Keywords:** crystal structure, sulfonate, hydrogen bond, π-stacking

## Abstract

In the title compound, C_26_H_18_N_2_O_6_S_2_, the pendant substituted *para-*toluene moieties are inclined relative to the central naphthalene-2,3-dicarbo­nitrile unit by 45.82 (7) and 42.41 (6)°. In the extended structure, the mol­ecules are linked by offset parallel π–π stacking and C—H⋯π inter­actions to form chains that propagate along the crystallographic *b-*axis direction. There are no conventional hydrogen bonds according to the usual distance and angle criteria, but a Hirshfeld surface analysis shows various contacts shorter than the van der Waals radii sums of the atoms concerned. The top four contributors to the packing have percentage contributions of 26.8, 26.0, 18.7 and 17.9% for H⋯H, H⋯O, H⋯C and H⋯N, respectively.

## Abstract 2

In the title compound, the pendant *para* toluene moieties are inclined to the central naphthalene-2,3-dicarbo­nitrile grouping by 45.82 (7) and 42.41 (6)°. The crystal packing features offset parallel π–π stacking and C—H⋯π inter­actions.

## 1. Chemical context

Aryl sulfonate derivatives show inter­esting supra­molecular behaviour in the solid state (El-Gamal *et al.*, 2020 ▸). The sulfonate ester functional group is comprised of two double-bonded oxygen atoms, a single-bonded oxygen atom and carbon atom creating a polar group capable of engaging in directional inter­molecular inter­actions (Côté & Shimizu, 2003 ▸; Korkmaz & Bursal, 2022 ▸). A number of aryl sulfonate derivatives are present in functional materials, synthetic inter­mediates and mol­ecular-recognition studies (Ghazzali *et al.*, 2013 ▸; Simpson & Widlanski, 2006 ▸; Hodges *et al.*, 2006 ▸). The effect of substituents on the resultant aryl sulfonate derivatives may significantly influence the conformational behaviour, planarity and rotation of the mol­ecules, thus determining the overall crystal packing efficiency. As part of our studies in this area, we now report the synthesis and structure of the title compound, C_26_H_18_N_2_O_6_S_2_ (**I**).
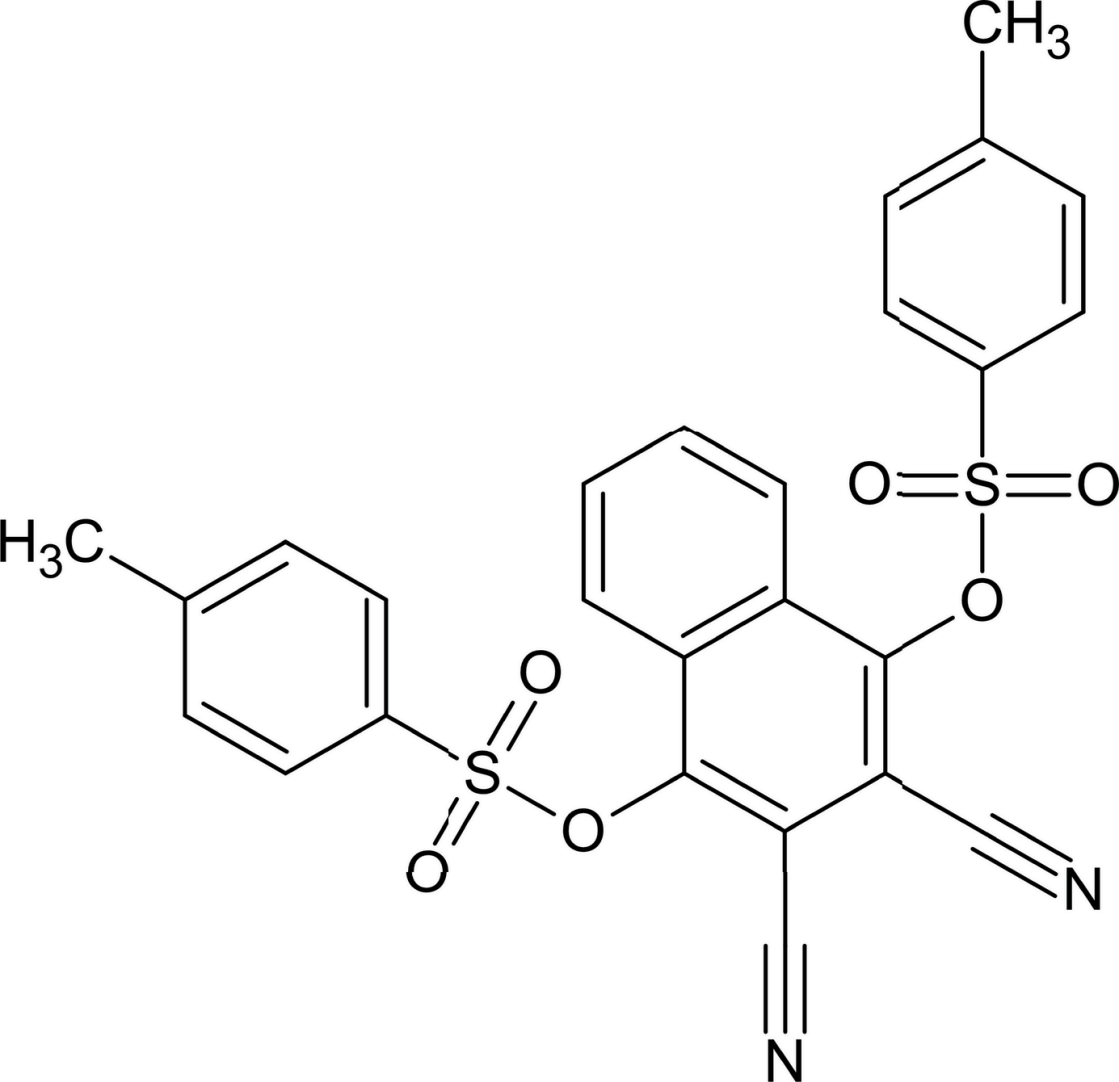


## 2. Structural commentary

**Figure 1:**
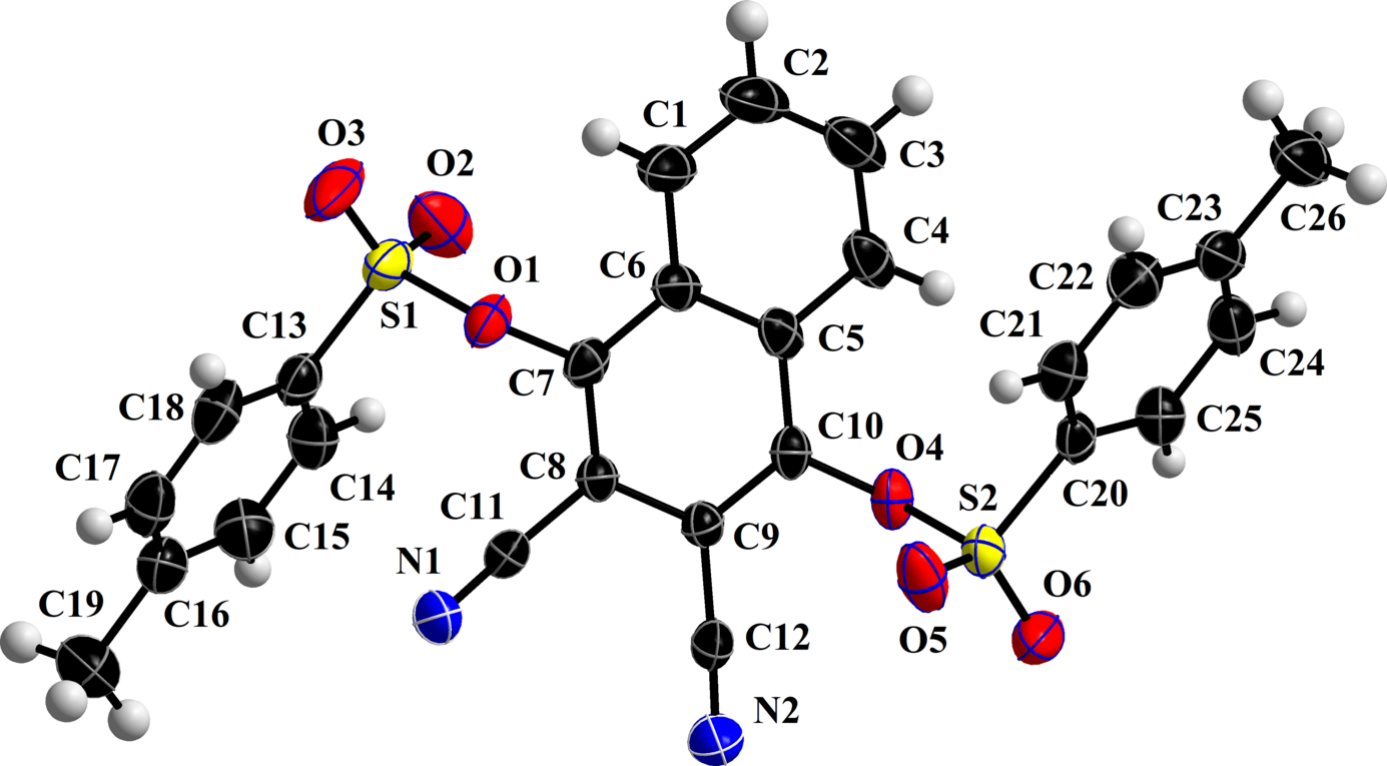
The mol­ecular structure of (**I**) showing 50% probability ellipsoids.

The crystal structure contains one mol­ecule of (**I**) in the asymmetric unit (Fig. 1 ▸) in space group *P*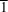. As expected, the central part of the mol­ecule (C1–C12/N1/N2) is close to planar with a root-mean-square deviation of 0.042 Å. The mol­ecule adopts a conformation such that the central part is inclined at dihedral angles of 45.82 (7) and 42.41 (6)°, respectively, relative to the *para* toluene moieties (C13–C19) and (C20–C26): one lies above the central plane and one lies below. The dihedral angle between the substituted *para* toluene moieties is 4.30 (18)°. The C7—O1—S1—C13 and C10—O4—S2—C20 torsion angles are 91.4 (2) and −94.6 (2)°, respectively. The distorted tetra­hedral geometry around the sulfur atoms in both sulfonate groups is very similar with the O=S=O bond angle [121.46 (18)° for S1 and 121.34 (14)° for S2] the largest in each case.

## 3. Supra­molecular features

**Figure 2:**
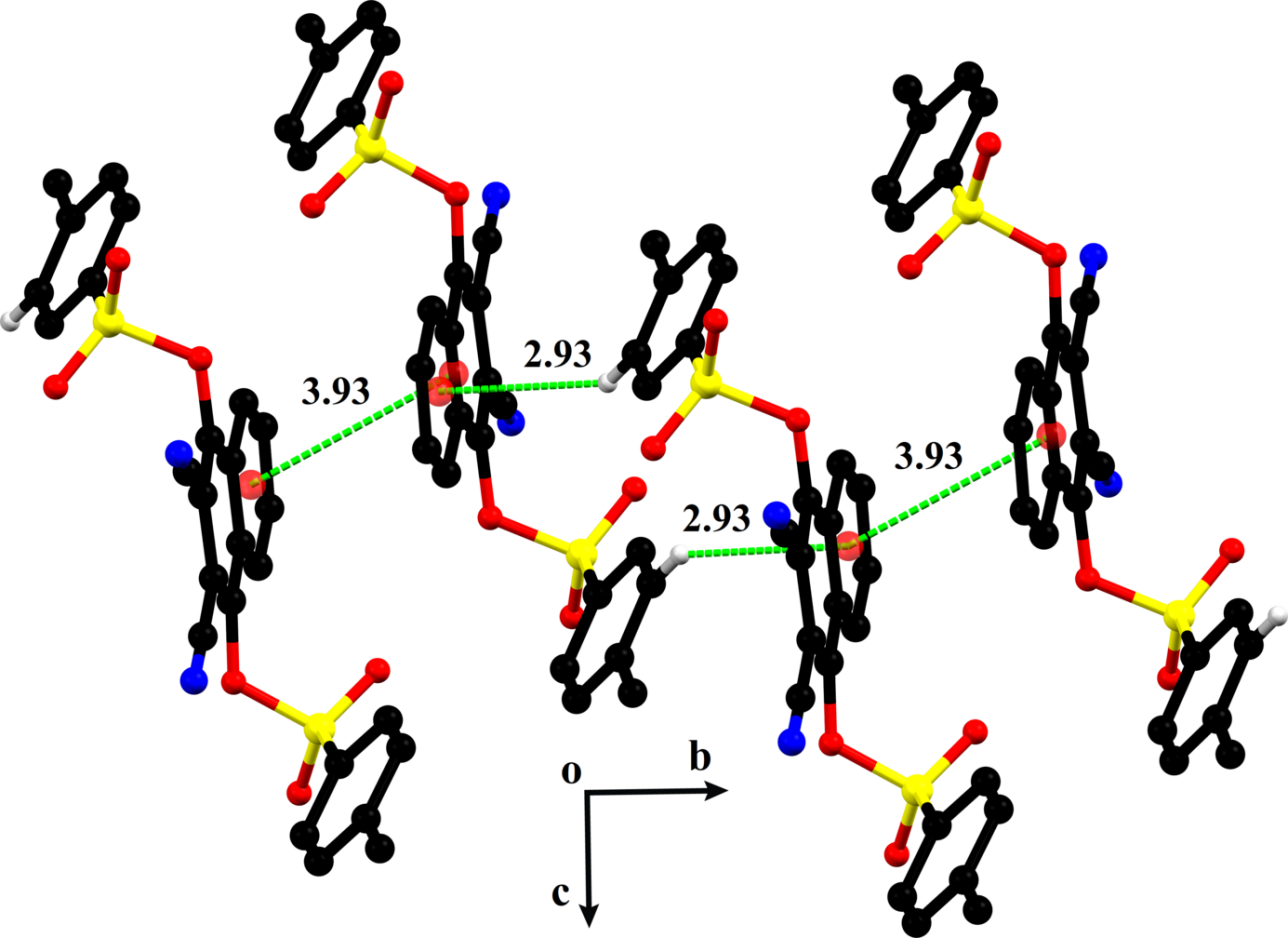
Fragment of a chain chain in the extended structure of (**I**) formed by offset parallel π–π stacking and C—H⋯π inter­actions. Distances shown are given in Å.

In the extended structure of (**I**), no hydrogen-bonding inter­actions are observed based on the standard distance and angle criteria for such inter­actions but see below. The mol­ecules are connected to each other through off-set parallel π–π stacking and C—H⋯π inter­actions with an inter-centroid distance of 3.9309 (15) Å and H⋯π distance of 2.93 Å. The value of the slippage distance for the offset parallel π–π stacking inter­action is 1.715 Å. As the results of these inter­actions, a supra­molecular chain is formed that propagates along the crystallographic *b*-axis direction (Fig. 2 ▸).

## 4. Hirshfeld surface analysis

**Figure 3:**
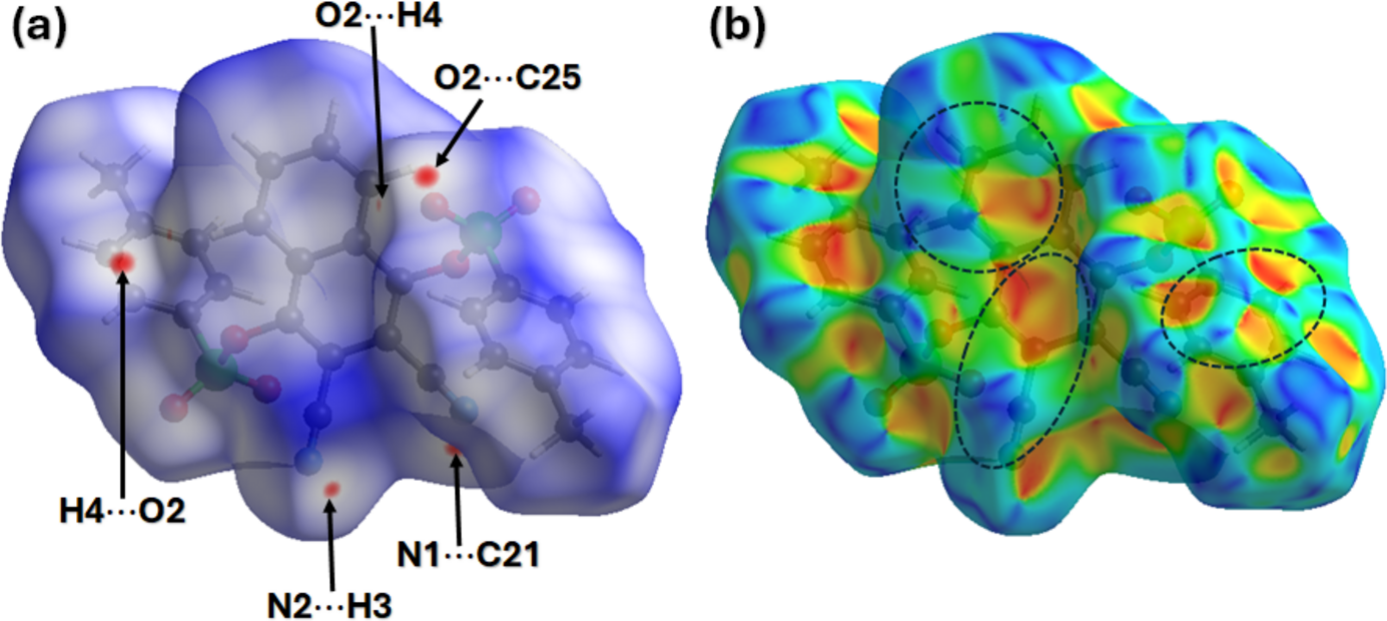
The Hirshfeld surface of (**I**) plotted over (*a*) *d*_norm_ and (*b*) shape-index. Short contacts associated with red spots in the *d*_norm_ plot include O2⋯H2 (2.69 Å), O2⋯C25 (3.15 Å), N2⋯H3 (2.69 Å) and N1⋯C21 (3.41 Å).

**Figure 4:**
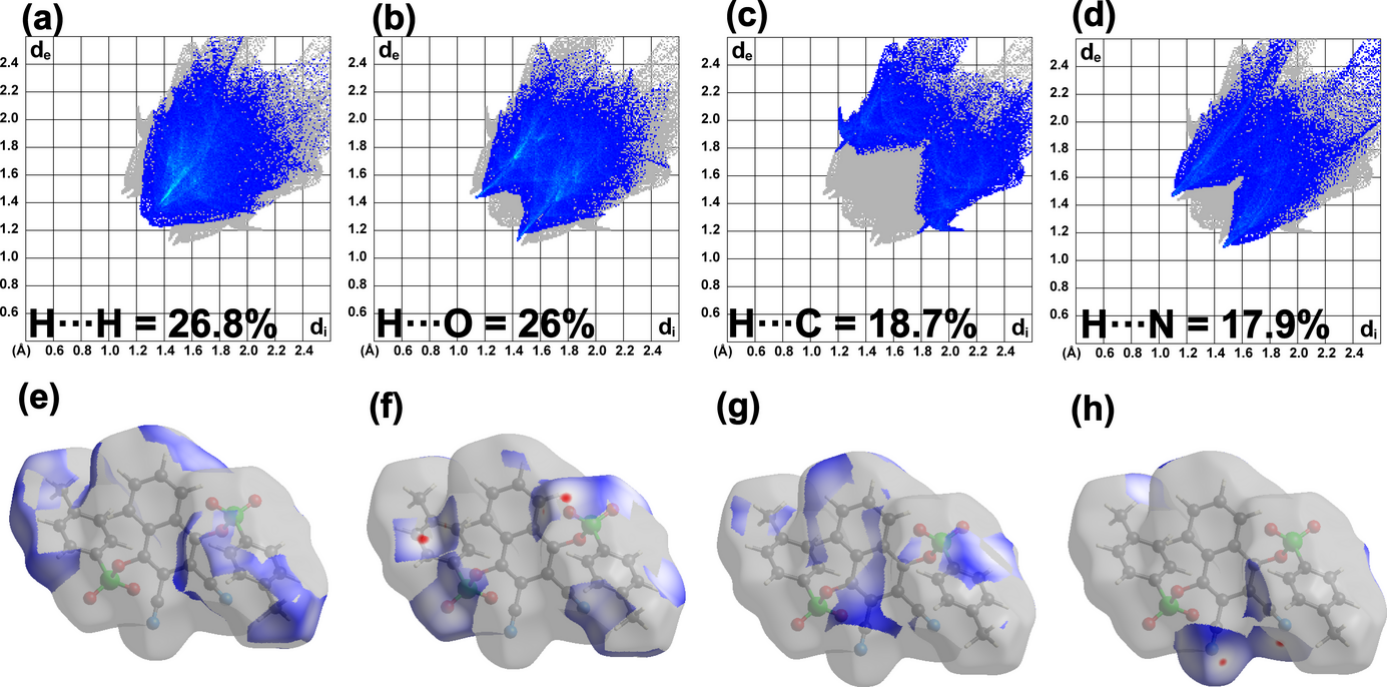
The two-dimensional fingerprint plots for (**I**) showing: (*a*)–(*d*) the top four contributing percentage contacts and (*e*)–(*h*) associated Hirshfeld surfaces.

A Hirshfeld surface (HS) analysis was carried out using *Crystal Explorer 21.5* (Spackman *et al.*, 2021 ▸). Fig. 3 ▸*a* shows the Hirshfeld surface plotted over *d*_norm_, normalized distances. Red spots on the surface around an O atom of the sulfonyl group, N atoms of cyano groups, and a CH group indicate that these atoms form short-range contacts. The face-to face red and blue triangular-shaped regions/patches on the surface of the shape index plot (Fig. 3 ▸*b*) around the aromatic rings indicate that weak π–π inter­actions are present in the crystal packing (Fig. 3 ▸*b*). The two-dimensional fingerprint plots show that H⋯H, H⋯O, H⋯C and H⋯N contacts make the largest contributions to the packing of 26.8%, 26%, 18.7% and 17.9%, respectively (Fig. 4 ▸*a*–*d*).

## 5. Database survey

A survey of the Cambridge Structural Database (CSD 6.0.1 updated November 2025; Groom *et al.*, 2015 ▸) found two structures having sulfonate groups attached to a naphthalene fused-ring system, *viz*. CSD refcodes HUSYAX (Hassan *et al.*, 2015 ▸) and WUFYAX (Yang *et al.*, 2002 ▸). Three polymorphs of the central naphthalene-2,3-dicarbo­nitrile group in the present compound have also been published [XEJNOV (Janczak & Kubiak, 2000 ▸), XEJNOV01 (Pitchumony & Stoeckli-Evans, 2005 ▸) and XEJNOV02 (Marsh, 2005 ▸)].

The bond lengths and bond angles of the present structure are consistent with corresponding ones in above-mentioned reported structures. The geometry around the sulfur atoms in HUSYAX and WUFYAX is distorted tetra­hedral, just as observed in the present structure. The central part of the mol­ecule in HUSYAX and WUFYAX is almost planar, with r.m.s. deviations of 0.042 and 0.043 Å, respectively.

## 6. Inter­action energy calculations and energy frameworks

The calculation of the inter­molecular inter­action energies using *Crystal Explorer* (Spackman *et al.*, 2021 ▸) provides a qu­anti­tative understanding of the types of forces that contribute to the aggregation of mol­ecules in the extended structure. By decomposing the overall inter­molecular inter­action energy into the electrostatic, polarization, dispersive, and repulsion components, the identification of dominant stabilizing inter­molecular inter­actions is now possible (Mackenzie *et al.*, 2017 ▸; Turner *et al.*, 2014 ▸). The maximum attractive inter­action for (**I**) occurs when the centroid-to-centroid distance between the mol­ecular pair is 5.71 Å with a total energy (*E*_total_) of −68.6 kJ mol^−1^ (Table S1). The total energy of the inter­action is dominated by a dispersion component (*E*_disp_ = −87.6 kJ mol^−1^), significantly larger than its repulsion contribution (*E*_rep_ = +43.3 kJ mol^−1^). Other close stabilizing contacts at centroid-to-centroid distances of 6.82, 8.11, and 9.98 Å are seen, with total energies of −53.7, −49.8 and −45.5 kJ mol^−1^, respectively, exhibiting complementary electrostatic and dispersion contributions. The energy framework plots of the crystal structure demonstrate that the dispersion energy contributes the most to the overall energy of the crystal structure as shown in the supporting information (Fig. S2*a*–*c*).

## 7. Synthesis and crystallization

**Figure 5:**
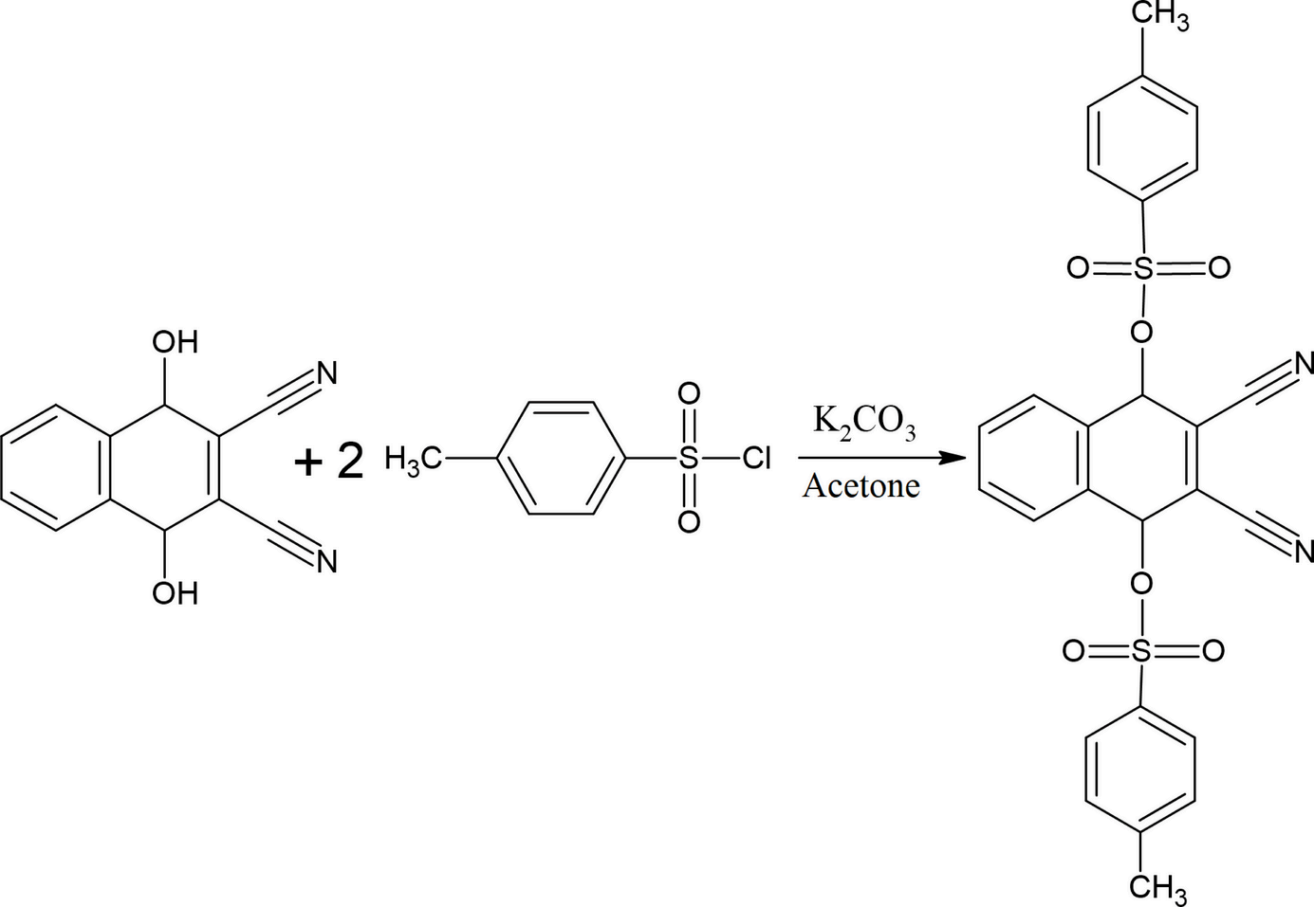
Reaction scheme.

4-Methyl­benzene-1-sulfonyl chloride (2.04 g, 9.71 mmol), 1,4-di­hydroxy­naphthalene-2,3-dicarbo­nitrile (4.39 g, 23.0 mmol) and K_2_CO_3_ (6 g, 43 mmol) in acetone (150 ml) were refluxed for 5 h and stirred under a nitro­gen atmosphere (Fig. 5 ▸). The reaction mixture was cooled and poured into ice–water (250 g). The product was filtered off and washed with 10% (*w*/*w*) NaOH solution and water until the filtrate was neutral and dried: yield = 5.04 g, 85.2%, m.p. 463 K. Single crystals of (**I**) in the form of orange blocks were obtained from aceto­nitrile solution at room temperature by slow evaporation. FT-IR (cm^−1^): 3053 (aromatic CH), 2977 (aliphatic CH), 2236 (C≡N) 1358 (sulfonyl ν_asym_) and 1175 (sulfonyl ν_sym_) (Fig. S3).

## 8. Refinement

Crystal data, data collection and structure refinement details are summarized in Table 1 ▸. The hydrogen atom positions were calculated geometrically at distances of 0.93 Å (for aromatic CH) and 0.96 Å (for CH_3_) and refined using a riding model with *U*_iso_(H) = 1.2*U*_eq_(C) or 1.5*U*_eq_(methyl C). 

## Supplementary Material

Crystal structure: contains datablock(s) I. DOI: 10.1107/S2056989026002884/hb8198sup1.cif

Structure factors: contains datablock(s) I. DOI: 10.1107/S2056989026002884/hb8198Isup2.hkl

Supporting information file. DOI: 10.1107/S2056989026002884/hb8198sup4.docx

Supporting information file. DOI: 10.1107/S2056989026002884/hb8198Isup4.cml

CCDC reference: 2539097

Additional supporting information:  crystallographic information; 3D view; checkCIF report

## Figures and Tables

**Table 1:** Experimental details

Crystal data	
Chemical formula	C_26_H_18_N_2_O_6_S_2_
*M*_r_	518.54
Crystal system, space group	Triclinic, *P* 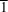
Temperature (K)	293
*a*, *b*, *c* (Å)	9.9829 (7), 10.8324 (8), 12.6366 (10)
α, β, γ (°)	108.467 (3), 90.006 (3), 111.812 (2)
*V* (Å^3^)	1192.53 (16)
*Z*	2
Radiation type	Mo *K*α
μ (mm^−1^)	0.27
Crystal size (mm)	0.21 × 0.17 × 0.15

Data collection	
Diffractometer	Bruker APEXII CCD
Absorption correction	Multi-scan (*SADABS*; Krause *et al.*, 2015 ▸)
*T*_min_, *T*_max_	0.619, 0.745
No. of measured, independent and observed [*I* > 2σ(*I*)] reflections	29617, 4856, 4032
*R*_int_	0.049
(sin θ/λ)_max_ (Å^−1^)	0.627

Refinement	
*R*[*F*^2^ > 2σ(*F*^2^)], *wR*(*F*^2^), *S*	0.057, 0.126, 1.16
No. of reflections	4856
No. of parameters	328
H-atom treatment	H-atom parameters constrained
Δρ_max_, Δρ_min_ (e Å^−3^)	0.36, −0.30

Computer programs: *APEX2* and *SAINT* (Bruker, 2014 ▸), *SHELXT2014/5* (Sheldrick, 2015*a* ▸), *SHELXL2025/1* (Sheldrick, 2015*b* ▸), *Mercury* (Macrae *et al.*, 2020 ▸) and *DIAMOND* (Brandenburg & Putz, 2005 ▸).

## supplementary crystallographic information

### 2,3-Dicyanonaphthalene-1,4-diyl bis(4-methylbenzene-1-sulfonate) Crystal data

**Table:**

C_26_H_18_N_2_O_6_S_2_	*Z* = 2
*M**_r_* = 518.54	*F*(000) = 536
Triclinic, *P*1	*D*_x_ = 1.444 Mg m^−^^3^
*a* = 9.9829 (7) Å	Mo *K*α radiation, λ = 0.71073 Å
*b* = 10.8324 (8) Å	Cell parameters from 4032 reflections
*c* = 12.6366 (10) Å	θ = 3.0–26.5°
α = 108.467 (3)°	µ = 0.27 mm^−^^1^
β = 90.006 (3)°	*T* = 293 K
γ = 111.812 (2)°	Block, orange
*V* = 1192.53 (16) Å^3^	0.21 × 0.17 × 0.15 mm

### 2,3-Dicyanonaphthalene-1,4-diyl bis(4-methylbenzene-1-sulfonate) Data collection

**Table:**

Bruker APEXII CCD diffractometer	4032 reflections with *I* > 2σ(*I*)
Detector resolution: 0.7979 pixels mm^-1^	*R*_int_ = 0.049
ω scans	θ_max_ = 26.5°, θ_min_ = 3.0°
Absorption correction: multi-scan (SADABS; Krause *et al.*, 2015)	*h* = −12→12
*T*_min_ = 0.619, *T*_max_ = 0.745	*k* = −13→13
29617 measured reflections	*l* = −15→15
4856 independent reflections	

### 2,3-Dicyanonaphthalene-1,4-diyl bis(4-methylbenzene-1-sulfonate) Refinement

**Table:**

Refinement on *F*^2^	Hydrogen site location: inferred from neighbouring sites
Least-squares matrix: full	H-atom parameters constrained
*R*[*F*^2^ > 2σ(*F*^2^)] = 0.057	*w* = 1/[σ^2^(*F*_o_^2^) + (0.0197*P*)^2^ + 1.3252*P*] where *P* = (*F*_o_^2^ + 2*F*_c_^2^)/3
*wR*(*F*^2^) = 0.126	(Δ/σ)_max_ < 0.001
*S* = 1.16	Δρ_max_ = 0.36 e Å^−^^3^
4856 reflections	Δρ_min_ = −0.30 e Å^−^^3^
328 parameters	Extinction correction: SHELXL-2025/1 (Sheldrick 2015b), Fc^*^=kFc[1+0.001xFc^2^λ^3^/sin(2θ)]^-1/4^
0 restraints	Extinction coefficient: 0.049 (2)
Primary atom site location: dual	

### 2,3-Dicyanonaphthalene-1,4-diyl bis(4-methylbenzene-1-sulfonate) Special details

**Table:**

Geometry. All esds (except the esd in the dihedral angle between two l.s. planes) are estimated using the full covariance matrix. The cell esds are taken into account individually in the estimation of esds in distances, angles and torsion angles; correlations between esds in cell parameters are only used when they are defined by crystal symmetry. An approximate (isotropic) treatment of cell esds is used for estimating esds involving l.s. planes.

### 2,3-Dicyanonaphthalene-1,4-diyl bis(4-methylbenzene-1-sulfonate) Fractional atomic coordinates and isotropic or equivalent isotropic displacement parameters (Å^2^)

**Table:**

	*x*	*y*	*z*	*U*_iso_*/*U*_eq_	
S1	0.38243 (8)	0.62969 (7)	0.88565 (6)	0.0516 (2)	
S2	0.14200 (7)	0.02617 (7)	0.30348 (6)	0.03969 (19)	
O1	0.3124 (2)	0.45839 (18)	0.81957 (14)	0.0408 (4)	
O2	0.4683 (2)	0.6927 (2)	0.8127 (2)	0.0718 (7)	
O3	0.4454 (3)	0.6399 (3)	0.9900 (2)	0.0881 (9)	
O4	0.21202 (19)	0.20023 (17)	0.35525 (14)	0.0371 (4)	
O5	0.0730 (2)	−0.0232 (2)	0.38872 (18)	0.0548 (5)	
O6	0.0617 (2)	−0.0017 (2)	0.20035 (17)	0.0605 (6)	
N1	−0.0626 (2)	0.3710 (3)	0.75159 (19)	0.0478 (6)	
N2	−0.1281 (3)	0.2042 (3)	0.4258 (2)	0.0618 (7)	
C1	0.5529 (3)	0.4413 (3)	0.7005 (3)	0.0530 (8)	
H1	0.571033	0.481170	0.778571	0.064*	
C2	0.6637 (3)	0.4311 (3)	0.6397 (3)	0.0650 (10)	
H2	0.757451	0.465769	0.676913	0.078*	
C3	0.6387 (3)	0.3697 (3)	0.5227 (3)	0.0610 (9)	
H3	0.716230	0.365652	0.482937	0.073*	
C4	0.5018 (3)	0.3153 (3)	0.4658 (3)	0.0465 (7)	
H4	0.486208	0.273185	0.387834	0.056*	
C5	0.3839 (3)	0.3232 (2)	0.5255 (2)	0.0344 (5)	
C6	0.4107 (3)	0.3910 (2)	0.6445 (2)	0.0361 (6)	
C7	0.2924 (3)	0.4036 (2)	0.7029 (2)	0.0327 (5)	
C8	0.1547 (2)	0.3531 (2)	0.64783 (19)	0.0295 (5)	
C9	0.1278 (2)	0.2831 (2)	0.52832 (19)	0.0298 (5)	
C10	0.2402 (3)	0.2674 (2)	0.47140 (19)	0.0309 (5)	
C11	0.0351 (3)	0.3641 (3)	0.70744 (19)	0.0336 (5)	
C12	−0.0152 (3)	0.2354 (3)	0.4705 (2)	0.0389 (6)	
C13	0.2307 (3)	0.6736 (3)	0.9029 (2)	0.0401 (6)	
C14	0.1866 (3)	0.7227 (3)	0.8263 (2)	0.0456 (6)	
H14	0.238631	0.734739	0.766786	0.055*	
C15	0.0644 (3)	0.7532 (3)	0.8397 (2)	0.0497 (7)	
H15	0.034337	0.786661	0.788666	0.060*	
C16	−0.0151 (3)	0.7352 (3)	0.9278 (2)	0.0456 (6)	
C17	0.0333 (3)	0.6884 (3)	1.0036 (2)	0.0500 (7)	
H17	−0.017879	0.677738	1.063739	0.060*	
C18	0.1554 (3)	0.6570 (3)	0.9928 (2)	0.0472 (7)	
H18	0.186455	0.625412	1.044743	0.057*	
C19	−0.1493 (4)	0.7677 (4)	0.9419 (3)	0.0708 (10)	
H19A	−0.122396	0.865196	0.987111	0.106*	
H19B	−0.214557	0.708540	0.978208	0.106*	
H19C	−0.196601	0.749963	0.869335	0.106*	
C20	0.2954 (3)	−0.0147 (2)	0.2792 (2)	0.0332 (5)	
C21	0.3516 (3)	−0.0549 (3)	0.3559 (2)	0.0424 (6)	
H21	0.307498	−0.062599	0.419600	0.051*	
C22	0.4745 (3)	−0.0835 (3)	0.3364 (2)	0.0482 (7)	
H22	0.513505	−0.110111	0.388143	0.058*	
C23	0.5413 (3)	−0.0737 (3)	0.2417 (2)	0.0453 (7)	
C24	0.4815 (3)	−0.0337 (3)	0.1663 (2)	0.0463 (7)	
H24	0.525008	−0.026795	0.102164	0.056*	
C25	0.3588 (3)	−0.0036 (3)	0.1834 (2)	0.0400 (6)	
H25	0.319824	0.023477	0.132009	0.048*	
C26	0.6732 (3)	−0.1085 (4)	0.2196 (3)	0.0671 (10)	
H26A	0.643189	−0.206686	0.175093	0.101*	
H26B	0.726899	−0.089700	0.289921	0.101*	
H26C	0.733834	−0.051388	0.179810	0.101*	

### 2,3-Dicyanonaphthalene-1,4-diyl bis(4-methylbenzene-1-sulfonate) Atomic displacement parameters (Å^2^)

**Table:**

	*U*^11^	*U*^22^	*U*^33^	*U*^12^	*U*^13^	*U*^23^
S1	0.0441 (4)	0.0375 (4)	0.0543 (4)	0.0164 (3)	−0.0115 (3)	−0.0095 (3)
S2	0.0377 (3)	0.0320 (3)	0.0413 (4)	0.0144 (3)	0.0068 (3)	0.0012 (3)
O1	0.0504 (11)	0.0338 (9)	0.0321 (9)	0.0182 (8)	−0.0060 (8)	0.0015 (7)
O2	0.0510 (13)	0.0369 (11)	0.111 (2)	0.0110 (10)	0.0247 (13)	0.0111 (12)
O3	0.0908 (18)	0.0875 (18)	0.0627 (15)	0.0547 (16)	−0.0434 (13)	−0.0281 (13)
O4	0.0487 (10)	0.0309 (9)	0.0323 (9)	0.0191 (8)	0.0112 (7)	0.0075 (7)
O5	0.0551 (12)	0.0371 (10)	0.0689 (14)	0.0163 (9)	0.0323 (10)	0.0159 (9)
O6	0.0549 (12)	0.0638 (14)	0.0504 (12)	0.0328 (11)	−0.0119 (10)	−0.0080 (10)
N1	0.0406 (13)	0.0582 (15)	0.0376 (12)	0.0188 (11)	0.0093 (10)	0.0080 (11)
N2	0.0503 (15)	0.085 (2)	0.0430 (14)	0.0363 (14)	−0.0056 (12)	0.0020 (13)
C1	0.0392 (15)	0.0435 (16)	0.067 (2)	0.0204 (13)	−0.0100 (13)	0.0017 (14)
C2	0.0344 (15)	0.0498 (18)	0.101 (3)	0.0201 (14)	−0.0056 (16)	0.0093 (18)
C3	0.0362 (15)	0.0443 (17)	0.099 (3)	0.0195 (13)	0.0195 (16)	0.0151 (17)
C4	0.0408 (15)	0.0314 (13)	0.0658 (18)	0.0172 (11)	0.0188 (13)	0.0112 (12)
C5	0.0343 (12)	0.0228 (11)	0.0469 (14)	0.0146 (10)	0.0089 (11)	0.0087 (10)
C6	0.0331 (12)	0.0249 (11)	0.0483 (14)	0.0145 (10)	0.0001 (10)	0.0063 (10)
C7	0.0380 (13)	0.0236 (11)	0.0338 (12)	0.0127 (10)	−0.0020 (10)	0.0055 (9)
C8	0.0309 (12)	0.0254 (11)	0.0317 (12)	0.0131 (9)	0.0036 (9)	0.0068 (9)
C9	0.0314 (12)	0.0277 (11)	0.0299 (12)	0.0136 (9)	0.0025 (9)	0.0070 (9)
C10	0.0393 (13)	0.0237 (11)	0.0309 (12)	0.0151 (10)	0.0068 (10)	0.0075 (9)
C11	0.0346 (13)	0.0328 (12)	0.0270 (12)	0.0117 (10)	−0.0002 (10)	0.0039 (9)
C12	0.0422 (15)	0.0470 (15)	0.0263 (12)	0.0239 (12)	0.0035 (10)	0.0033 (11)
C13	0.0450 (14)	0.0307 (12)	0.0351 (13)	0.0135 (11)	−0.0024 (11)	0.0003 (10)
C14	0.0534 (16)	0.0439 (15)	0.0398 (14)	0.0196 (13)	0.0122 (12)	0.0141 (12)
C15	0.0597 (18)	0.0547 (17)	0.0454 (16)	0.0299 (15)	0.0071 (13)	0.0221 (14)
C16	0.0461 (15)	0.0416 (15)	0.0433 (15)	0.0177 (12)	0.0050 (12)	0.0065 (12)
C17	0.0592 (18)	0.0486 (16)	0.0367 (14)	0.0189 (14)	0.0118 (13)	0.0103 (12)
C18	0.0659 (19)	0.0398 (15)	0.0320 (13)	0.0206 (14)	−0.0026 (12)	0.0072 (11)
C19	0.056 (2)	0.089 (3)	0.069 (2)	0.0377 (19)	0.0122 (17)	0.0162 (19)
C20	0.0370 (13)	0.0243 (11)	0.0332 (12)	0.0124 (10)	0.0069 (10)	0.0028 (9)
C21	0.0511 (16)	0.0391 (14)	0.0357 (14)	0.0167 (12)	0.0077 (11)	0.0122 (11)
C22	0.0538 (17)	0.0423 (15)	0.0497 (16)	0.0232 (13)	−0.0022 (13)	0.0120 (13)
C23	0.0364 (14)	0.0287 (13)	0.0586 (17)	0.0113 (11)	0.0024 (12)	0.0010 (12)
C24	0.0496 (16)	0.0408 (15)	0.0457 (15)	0.0186 (13)	0.0178 (13)	0.0100 (12)
C25	0.0465 (15)	0.0377 (14)	0.0367 (13)	0.0185 (12)	0.0089 (11)	0.0116 (11)
C26	0.0451 (17)	0.057 (2)	0.090 (3)	0.0273 (15)	0.0055 (16)	0.0045 (18)

### 2,3-Dicyanonaphthalene-1,4-diyl bis(4-methylbenzene-1-sulfonate) Geometric parameters (Å, º)

**Table:**

S1—O3	1.415 (2)	C13—C14	1.383 (4)
S1—O2	1.419 (3)	C13—C18	1.385 (4)
S1—O1	1.6382 (18)	C14—C15	1.375 (4)
S1—C13	1.743 (3)	C14—H14	0.9300
S2—O6	1.415 (2)	C15—C16	1.390 (4)
S2—O5	1.418 (2)	C15—H15	0.9300
S2—O4	1.6464 (17)	C16—C17	1.377 (4)
S2—C20	1.747 (2)	C16—C19	1.503 (4)
O1—C7	1.387 (3)	C17—C18	1.378 (4)
O4—C10	1.391 (3)	C17—H17	0.9300
N1—C11	1.138 (3)	C18—H18	0.9300
N2—C12	1.140 (3)	C19—H19A	0.9600
C1—C2	1.365 (4)	C19—H19B	0.9600
C1—C6	1.412 (3)	C19—H19C	0.9600
C1—H1	0.9300	C20—C21	1.375 (4)
C2—C3	1.394 (5)	C20—C25	1.385 (3)
C2—H2	0.9300	C21—C22	1.377 (4)
C3—C4	1.366 (4)	C21—H21	0.9300
C3—H3	0.9300	C22—C23	1.385 (4)
C4—C5	1.413 (3)	C22—H22	0.9300
C4—H4	0.9300	C23—C24	1.382 (4)
C5—C10	1.408 (3)	C23—C26	1.502 (4)
C5—C6	1.423 (4)	C24—C25	1.380 (4)
C6—C7	1.419 (3)	C24—H24	0.9300
C7—C8	1.368 (3)	C25—H25	0.9300
C8—C9	1.432 (3)	C26—H26A	0.9600
C8—C11	1.434 (3)	C26—H26B	0.9600
C9—C10	1.368 (3)	C26—H26C	0.9600
C9—C12	1.432 (3)		
			
O3—S1—O2	121.46 (18)	C14—C13—S1	119.7 (2)
O3—S1—O1	101.94 (13)	C18—C13—S1	119.1 (2)
O2—S1—O1	107.23 (12)	C15—C14—C13	118.9 (3)
O3—S1—C13	110.58 (15)	C15—C14—H14	120.6
O2—S1—C13	110.13 (14)	C13—C14—H14	120.6
O1—S1—C13	103.64 (11)	C14—C15—C16	121.4 (3)
O6—S2—O5	121.34 (14)	C14—C15—H15	119.3
O6—S2—O4	102.41 (12)	C16—C15—H15	119.3
O5—S2—O4	107.33 (10)	C17—C16—C15	118.2 (3)
O6—S2—C20	110.53 (12)	C17—C16—C19	120.4 (3)
O5—S2—C20	110.27 (13)	C15—C16—C19	121.4 (3)
O4—S2—C20	103.03 (10)	C16—C17—C18	121.9 (3)
C7—O1—S1	120.59 (16)	C16—C17—H17	119.0
C10—O4—S2	119.14 (15)	C18—C17—H17	119.0
C2—C1—C6	119.8 (3)	C17—C18—C13	118.4 (3)
C2—C1—H1	120.1	C17—C18—H18	120.8
C6—C1—H1	120.1	C13—C18—H18	120.8
C1—C2—C3	121.1 (3)	C16—C19—H19A	109.5
C1—C2—H2	119.5	C16—C19—H19B	109.5
C3—C2—H2	119.5	H19A—C19—H19B	109.5
C4—C3—C2	120.9 (3)	C16—C19—H19C	109.5
C4—C3—H3	119.6	H19A—C19—H19C	109.5
C2—C3—H3	119.6	H19B—C19—H19C	109.5
C3—C4—C5	119.9 (3)	C21—C20—C25	121.6 (2)
C3—C4—H4	120.1	C21—C20—S2	119.66 (19)
C5—C4—H4	120.1	C25—C20—S2	118.8 (2)
C10—C5—C4	122.3 (2)	C20—C21—C22	118.7 (2)
C10—C5—C6	118.5 (2)	C20—C21—H21	120.6
C4—C5—C6	119.1 (2)	C22—C21—H21	120.6
C1—C6—C7	122.3 (2)	C21—C22—C23	121.6 (3)
C1—C6—C5	119.1 (2)	C21—C22—H22	119.2
C7—C6—C5	118.6 (2)	C23—C22—H22	119.2
C8—C7—O1	118.1 (2)	C24—C23—C22	118.1 (3)
C8—C7—C6	121.8 (2)	C24—C23—C26	120.6 (3)
O1—C7—C6	119.9 (2)	C22—C23—C26	121.3 (3)
C7—C8—C9	119.5 (2)	C25—C24—C23	121.8 (3)
C7—C8—C11	121.6 (2)	C25—C24—H24	119.1
C9—C8—C11	118.9 (2)	C23—C24—H24	119.1
C10—C9—C8	119.3 (2)	C24—C25—C20	118.2 (3)
C10—C9—C12	121.4 (2)	C24—C25—H25	120.9
C8—C9—C12	119.3 (2)	C20—C25—H25	120.9
C9—C10—O4	118.4 (2)	C23—C26—H26A	109.5
C9—C10—C5	122.3 (2)	C23—C26—H26B	109.5
O4—C10—C5	119.2 (2)	H26A—C26—H26B	109.5
N1—C11—C8	177.9 (3)	C23—C26—H26C	109.5
N2—C12—C9	176.6 (3)	H26A—C26—H26C	109.5
C14—C13—C18	121.2 (3)	H26B—C26—H26C	109.5
			
O3—S1—O1—C7	−153.7 (2)	C4—C5—C10—C9	−175.7 (2)
O2—S1—O1—C7	−25.1 (2)	C6—C5—C10—C9	3.7 (3)
C13—S1—O1—C7	91.4 (2)	C4—C5—C10—O4	0.9 (3)
O6—S2—O4—C10	150.60 (18)	C6—C5—C10—O4	−179.7 (2)
O5—S2—O4—C10	21.8 (2)	O3—S1—C13—C14	155.5 (2)
C20—S2—O4—C10	−94.61 (18)	O2—S1—C13—C14	18.5 (3)
C6—C1—C2—C3	−1.0 (5)	O1—S1—C13—C14	−96.0 (2)
C1—C2—C3—C4	−1.3 (5)	O3—S1—C13—C18	−25.3 (3)
C2—C3—C4—C5	1.1 (4)	O2—S1—C13—C18	−162.3 (2)
C3—C4—C5—C10	−179.2 (3)	O1—S1—C13—C18	83.3 (2)
C3—C4—C5—C6	1.4 (4)	C18—C13—C14—C15	−0.9 (4)
C2—C1—C6—C7	−177.6 (3)	S1—C13—C14—C15	178.3 (2)
C2—C1—C6—C5	3.5 (4)	C13—C14—C15—C16	−0.3 (4)
C10—C5—C6—C1	176.9 (2)	C14—C15—C16—C17	1.3 (4)
C4—C5—C6—C1	−3.7 (4)	C14—C15—C16—C19	−179.5 (3)
C10—C5—C6—C7	−2.1 (3)	C15—C16—C17—C18	−1.2 (4)
C4—C5—C6—C7	177.3 (2)	C19—C16—C17—C18	179.6 (3)
S1—O1—C7—C8	−102.8 (2)	C16—C17—C18—C13	0.1 (4)
S1—O1—C7—C6	82.1 (2)	C14—C13—C18—C17	1.0 (4)
C1—C6—C7—C8	−179.2 (2)	S1—C13—C18—C17	−178.2 (2)
C5—C6—C7—C8	−0.3 (3)	O6—S2—C20—C21	−151.9 (2)
C1—C6—C7—O1	−4.3 (4)	O5—S2—C20—C21	−15.0 (2)
C5—C6—C7—O1	174.6 (2)	O4—S2—C20—C21	99.3 (2)
O1—C7—C8—C9	−173.8 (2)	O6—S2—C20—C25	29.1 (2)
C6—C7—C8—C9	1.2 (3)	O5—S2—C20—C25	166.04 (19)
O1—C7—C8—C11	4.0 (3)	O4—S2—C20—C25	−79.7 (2)
C6—C7—C8—C11	179.0 (2)	C25—C20—C21—C22	0.4 (4)
C7—C8—C9—C10	0.4 (3)	S2—C20—C21—C22	−178.5 (2)
C11—C8—C9—C10	−177.5 (2)	C20—C21—C22—C23	−0.4 (4)
C7—C8—C9—C12	−177.5 (2)	C21—C22—C23—C24	0.2 (4)
C11—C8—C9—C12	4.6 (3)	C21—C22—C23—C26	−178.5 (3)
C8—C9—C10—O4	−179.5 (2)	C22—C23—C24—C25	0.1 (4)
C12—C9—C10—O4	−1.6 (3)	C26—C23—C24—C25	178.8 (3)
C8—C9—C10—C5	−2.9 (3)	C23—C24—C25—C20	−0.2 (4)
C12—C9—C10—C5	175.0 (2)	C21—C20—C25—C24	−0.1 (4)
S2—O4—C10—C9	−82.1 (2)	S2—C20—C25—C24	178.83 (19)
S2—O4—C10—C5	101.2 (2)		
