# Supplementary material for: Hydrogen bonds or not? Synthesis and structure of 2,3-di­cyanona­phthalene-1,4-diyl bis­(4-methylbenzene-1-sulfonate)
Source: Acta Crystallogr E Crystallogr Commun. 2026 Mar 24;82(Pt 4):400–3. doi: 10.1107/S2056989026002884 (PMC13055959; doi:10.1107/S2056989026002884)
Supplement: Supplementary file 3 [file e-82-00400-sup4.docx]

**Supplementary File**


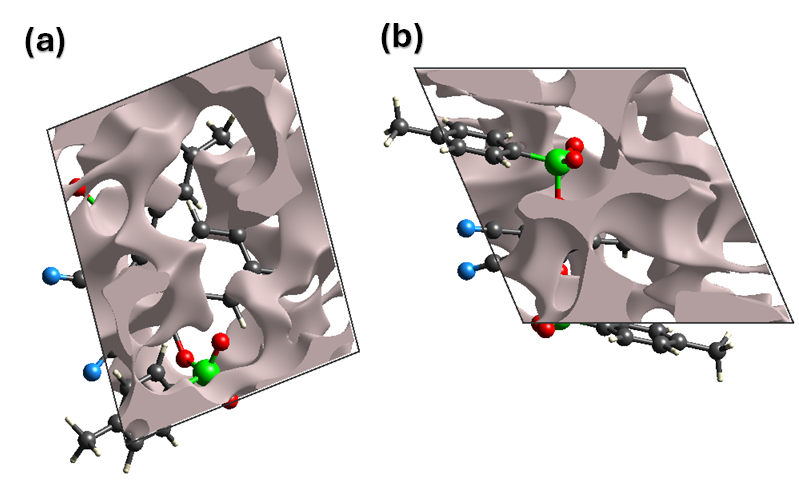


**Figure S1.** Isosurfaces of voids, two alternate views.


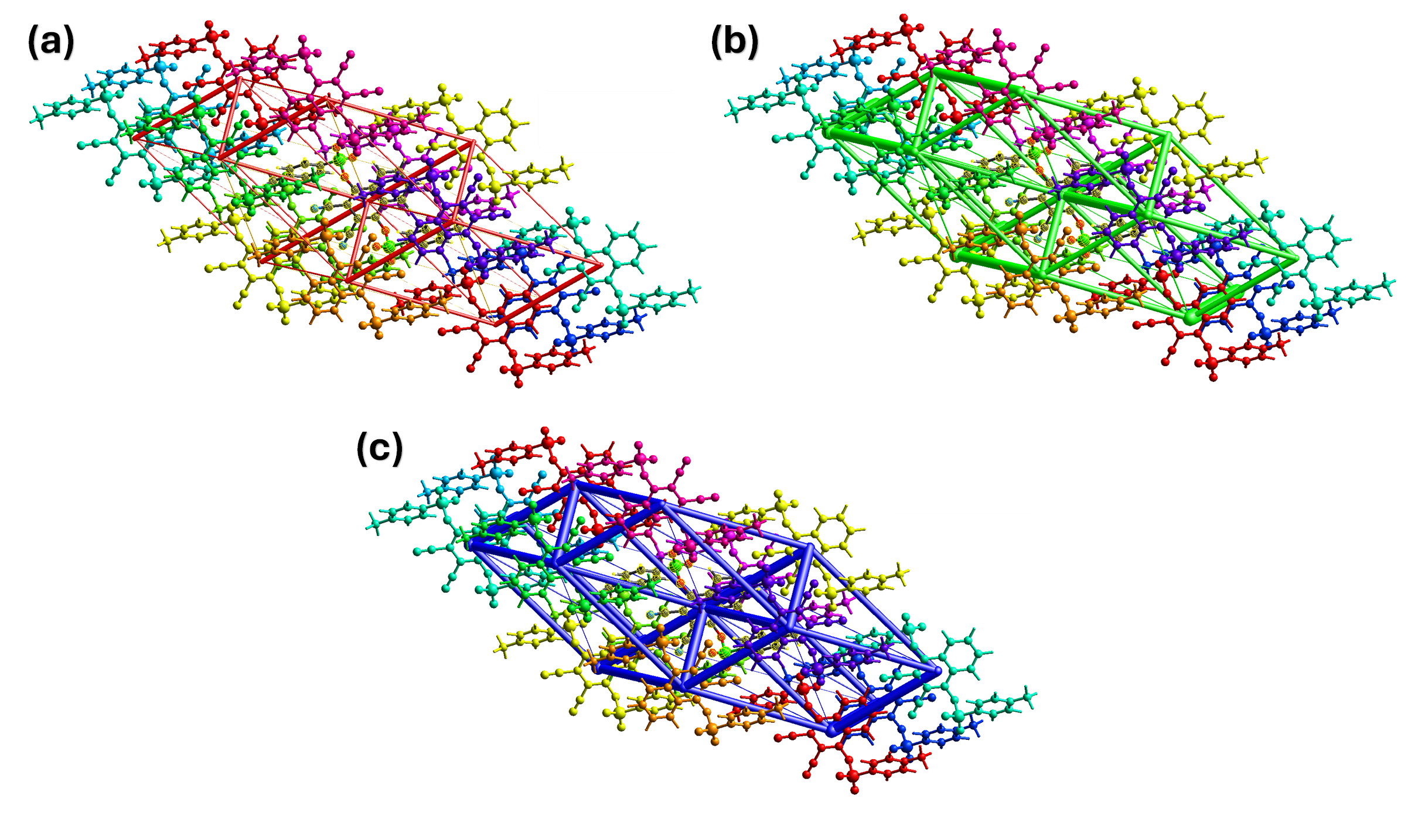


**Figure S2**. Energy frameworks for **(a)** coulomb energy, **(b)** dispersion energy, **(c)** total energy.

**Figure S3.** FT-IR spectrum of 2,3-dicyanonaphthalene-1,4-diyl bis(4-methylbenzenesulfonate).

**Figure S4.** UV-Vis spectrums of 2,3-dicyanonaphthalene-1,4-diyl bis(4-methylbenzenesulfonate) in various solvents

Table S1. Interaction Energies (kJ/mol) calculated at B3LYP/6-31G(d,p) electron density level. R is the distance between molecular centroids (mean atomic position) in Å.

Total energies, only reported for two benchmarked energy models, are the sum of the

four energy components, scaled appropriately (see the scale factor table below)

--------------------------------------------------------------------------------

|  | N | Symop | R | E_ele | E_pol | E_dis | E_rep | E_tot |
| --- | --- | --- | --- | --- | --- | --- | --- | --- |
|  | 2 | x, y, z | 13.80 | -7.3 | -3.7 | -28.2 | 0.0 | -35.1 |
|  | 1 | -x, -y, -z | 6.82 | -20.8 | -9.0 | -47.1 | 25.7 | -53.7 |
|  | 2 | x, y, z | 9.98 | -24.2 | -8.2 | -31.8 | 22.4 | -45.5 |
|  | 1 | -x, -y, -z | 8.11 | -22.1 | -9.2 | -40.4 | 25.2 | -49.8 |
|  | 1 | -x, -y, -z | 11.82 | -16.3 | -5.1 | -36.4 | 27.1 | -36.1 |
|  | 2 | x, y, z | 19.24 | 1.5 | -0.4 | -4.5 | 0.0 | -2.6 |
|  | 1 | -x, -y, -z | 17.52 | 0.7 | -0.3 | -5.5 | 0.0 | -4.3 |
|  | 1 | -x, -y, -z | 15.89 | -1.3 | -1.2 | -5.4 | 0.0 | -7.0 |
|  | 1 | -x, -y, -z | 9.68 | -7.5 | -4.7 | -59.6 | 27.4 | -46.4 |
|  | 1 | -x, -y, -z | 5.71 | -13.5 | -6.5 | -87.6 | 43.3 | -68.6 |
|  | 1 | -x, -y, -z | 10.32 | 4.1 | -1.4 | -8.5 | 0.5 | -3.8 |

--------------------------------------------------------------------------------

Scale factors for benchmarked energy models

See Mackenzie et al. IUCrJ (2017)

--------------------------------------------------------------------------------

| Energy Model | k_ele | k_pol | k_disp | k_rep |
| --- | --- | --- | --- | --- |
| CE-B3LYP ... B3LYP/6-31G(d,p) electron densities | 1.057 | 0.740 | 0.871 | 0.618 |
